# Modeling and Optimization of Process Parameters for Nutritional Enhancement in Enzymatic Milled Rice by Multiple Linear Regression (MLR) and Artificial Neural Network (ANN)

**DOI:** 10.3390/foods10122975

**Published:** 2021-12-03

**Authors:** Anjineyulu Kothakota, Ravi Pandiselvam, Kaliramesh Siliveru, Jai Prakash Pandey, Nukasani Sagarika, Chintada H. Sai Srinivas, Anil Kumar, Anupama Singh, Shivaprasad D. Prakash

**Affiliations:** 1 Agro-Processing & Technology Division, CSIR-National Institute for Interdisciplinary Science and Technology, Thiruvananthapuram 695019, Kerala, India; 2 Physiology, Biochemistry and Post-Harvest Technology Division, ICAR-Central Plantation Crops Research Institute, Chowki 671124, Kerala, India; anbupandi1989@yahoo.co.in; 3 Department of Grain Science & Industry, Kansas State University, Manhattan, KS 66502, USA; shivdp1994@ksu.edu; 4 Department of Post-Harvest Process and Food Engineering, College of Technology, G.B. Pant University of Agriculture and Technology, Pantnagar 263145, Uttarakhand, India; jppandey55@gmail.com (J.P.P.); asingh3@gmail.com (A.S.); 5 Department of Food Process Engineering, College of Food Processing Technology & Bio-Energy, Anand Agricultural University, Anand 388110, Gujarat, India; sagarikanukasani@gmail.com; 6 Foods Business Division, ITC Limited, Visakhapatnam 530001, Andhra Pradesh, India; saisrinivas36cfst@gmail.com; 7 Department of Food Science and Technology, College of Agriculture, G.B. Pant University of Agriculture and Technology, Pantnager 263145, India; anilkumargbpuat@gmail.com

**Keywords:** multiple linear regression (MLR), artificial neural network (ANN), milled rice, enzymes

## Abstract

This study involves information about the concentrations of nutrients (proteins, phenolic compounds, free amino acids, minerals (Ca, P, and Iron), hardness) in milled rice processed with enzymes; xylanase and cellulase produced by *Aspergillus awamori*, MTCC 9166 and *Trichoderma reese*, MTCC164. Brown rice was processed with 60–100% enzyme (40 mL buffer -undiluted) for 30 to 150 min at 30 °C to 50 °C followed by polishing for 20–100 s at a safe moisture level. Multiple linear regression (MLR) and artificial neural network (ANN) models were used for process optimization of enzymes. The MLR correlation coefficient (R^2^) varied between 0.87–0.90, and the sum of square (SSE) was placed within 0.008–8.25. While the ANN R^2^ (correlation coefficient) varied between 0.97 and 0.9999(1), MSE changed from 0.005 to 6.13 representing that the ANN method has better execution across MLR. The optimized cellulase process parameters (87.2% concentration, 80.1 min process time, 33.95 °C temperature and 21.8 s milling time) and xylanase process parameters (85.7% enzyme crude, 77.1 min process time, 35 °C temperature and 20 s) facilitated the increase of Ca (70%), P (64%), Iron (17%), free amino acids (34%), phenolic compounds (78%) and protein (84%) and decreased hardness (20%) in milled rice. Scanning electron micrographs showed an increased rupture attributing to enzymes action on milled rice.

## 1. Introduction

Rice (*Oryza sativa* L.) exists as an essential commodity all over the world. This must be a prominent subsistence grain for growing nations and a primary energy source containing bioactive compounds, vitamins, minerals, amino acids, and fiber [1]. Brown rice dehusked from paddy consists of an embryo (2–3% of its total weight), endosperm (90%), and bran layer (6–7%) [2]. The bran layer’s major constituents are protein, fat, crude fiber, ash, carbohydrates, cellulose, arabinoxylans, mannans, Galatians, pentosans, and uronic acid. It additionally furnishes a substantial quantity of B1, B2, B3, Zn, and smaller amounts of different trace elements [3].

The commercial value of rice grains is established over these proportions and moisture of crop; these characteristics further support more portion of fracture throughout automatic polishing. However, it may not be suitable for consumer consumption. Polishing the grain to a proper degree may be essential for preserving the quality and characteristics of the grain. Degrading the bran structure in paddy grains is a critical stage for the grain in an individual’s ingestion [4]. The loss of essential nutrients in case milling have been shown to occur through over-processing; reducing such losses throughout automatic milling may be conquered via novel pre-treatment: cold plasma [5], high-pressure processing [6], ultrasonication [7], pulsed electric field processing [8], and enzymes treatment [9], before polishing to improve the milling properties. We have attempted to apply enzymes as a pre-treatment to enhance the nutrient content after milling. Pretreatments are evidence to enhance seed functioning by affecting the biochemical and physiological qualities of grains without adversely affecting the atmosphere. Polishing is a primary treatment step for cereals to eliminate their hard-cellulosic cover and bran adhering to the surface. Several pretreatment methods, such as high hydrostatic pressure (HHP), cold plasma, pulse electric filed, UV light, microwave and enzymatic treatment can ensure the nutritional improvement in cereals and millets for shorter processing time at various conditions [9,10].

Some investigations have described how the complete breakdown of bran structure through different enzymes (amylase and glucanase) may improve the textural characteristics of milled rice due to the bran structure interface through polysaccharides (cellulose, hemicelluloses, and amylase) via various bonds; glycosidic, covalent, and hydrolytic bonds may be adhered and hydrolyzed with endoglucanase, cellulase, and xylanase [9,10]. These bonds may perform to limit bran structure decline during the degree of milling materials.

Considering the above factors, both Cellulase and xylanase enzymes, have been selected for the process of brown rice, improving the polishing nature, cooking characteristics, retaining the nutrients in polished rice, and textural characteristics. Few investigations have been carried out, but none have attempted to model and optimize processing conditions by using MLR (multiple linear regression) and ANN (artificial neural networks) to enhance nutritional properties in milled rice.

The modeling and optimization of treatment conditions for nutritional enhancement has had a problematic method, which has been analyzed as far as agriculture based products, food products, beverages, dairy products, and oil extraction industry products have pertained to different design methods to attain reasonable valuable resources [11]. Multiple linear regression (MLR) may be described while an experimental modeling design is employed as formulating, enhancing, and reducing complicated operations [12,13]. This method has the benefit of reducing the number of empirical tests and may be sufficient to provide a significantly acceptable outcome [13]. It is used for modeling and optimizes nutritional increments in food products: Extrusion [13,14], bakery foods [15], meat products [16], oils [17], and enzymes [18] are enzymatically treated during processing [19,20]. ANN sought tools and design for learning a simple process from the nonlinear association between input data to output data in a system compared to MLR. Lately, several researchers have mentioned ANN utilization in favor of optimizing conditions for food processing, including the enzymes production for beverages [11] and enzyme application in food [18]. This study aimed to (1) generate cellulase and xylanase enzymes with *Aspergillus awamori* (MTCC 9166) as well as *Trichoderma reesei* Rut C-30(MTCC16675) for the intent of processing, (2) modeling and optimizing enzyme-treated parameters for rice by applying MLR and ANN, and (3) to evaluate the nutritional and textural properties of enzyme treated milled rice.

## 2. Materials and Methods

### 2.1. Rice Samples 

The Pant Sugandh Dhan15 (Aromatic, long and slender) rough rice was acquired with a Crop Research Centre, GB Panth University of Agriculture and Technology, Pantnagar. The sample was stored in an airtight container to avert the moisture interchange with the atmosphere. Rough rice was dehusked for further experimentation.

All Reagents were acquired with Hi-media Laboratories Pvt Ltd., Mumbai, and Sigma–Aldrich, New Delhi, India.

### 2.2. Enzyme Preparation

**Figure 1:**
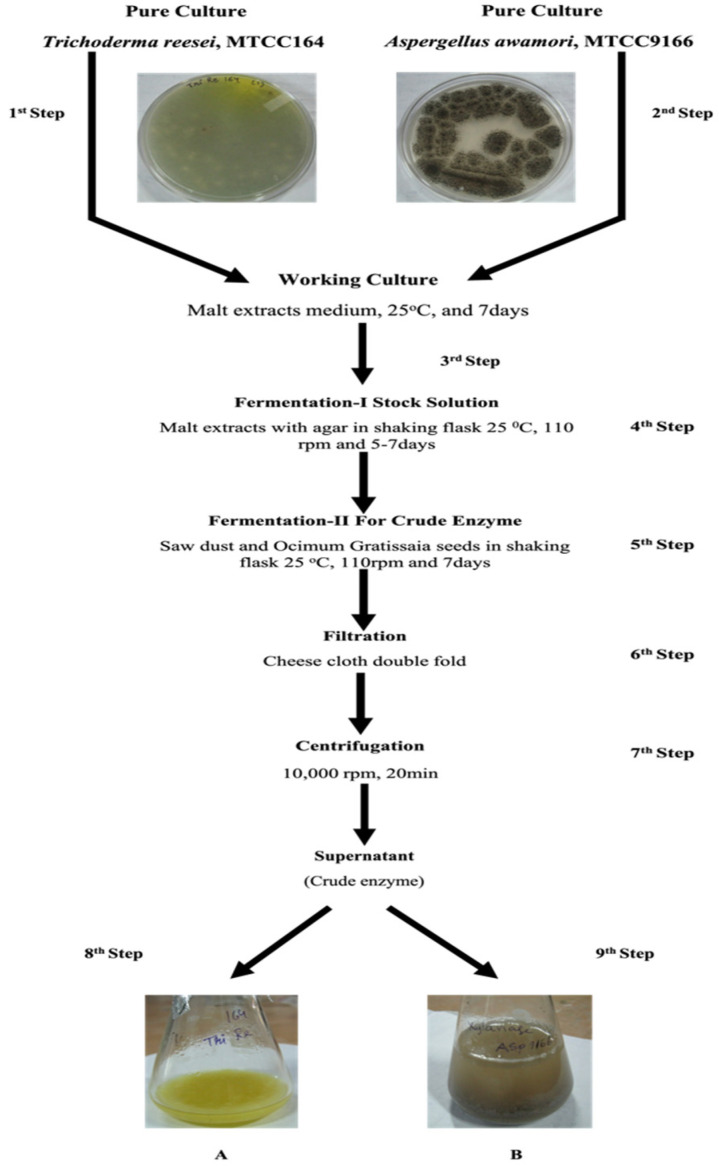
Flow diagram of cellulase (**A**) and xylanase (**B**) enzyme production.

The Fungal crude cellulase and xylanase were produced from *Trichoderma reesei* Rut C-30 (MTCC16675) and *Aspergillus awamori* (MTCC 9166) submerged fermentation (Figure 1) was used as the enzyme activity. Enzyme activities can be expressed in the enzyme unit (U). 1 U was determined as the quantity by which the transformation is induced of about one micromole of matrix materials per minute through the particular circumstances of the analysis method [9,20]. The produced enzyme were diluted with different ratios: 100% (undiluted), 90% (90 mL crude + 10 mL buffer), 80% (80 mL crude + 20 mL buffer), 70% (70 mL crude + 30 mL buffer), and 60% (60 mL crude + 40 mL buffer) [19,20].

### 2.3. Experimental Process 

The head brown rice (100 g) was soaked in 50 mL water for 24 h, and the water was altered at specific gaps of time to decrease microbial infection. Soaked grains were again treated to an additional one hour in 100 mL sterile water about 5 g of calcium carbonate on 55 °C to create calcium ions enforce as a promoter to the enzyme action. These soaked samples processed cellulase and xylanase at various concentrations in the ratio 100% to 60% appropriately, for process the brown rice at various temperatures 30 °C to 50 °C (with 5 °C variation) in distinct time: 30–150 min (with 30 min variation). The processed samples were polished at various times, 20–100 s (20 s variation) through an abrasive Satake polisher. After de-husking, the polished rice was removed by sieving [19,20].

#### 2.3.1. Estimation of Mineral Content

Determinations of mineral content in rice were measured by atomic absorption spectroscopy (Spectro Ciros C CD, Spectro, and Dusseldorf, Germany) in ppm according to [9]. 

#### 2.3.2. Total Phenolic Substance Estimation 

The phenolic content of milled rice was estimated through the Follin–Ciocalteu reagent method by spectrophotometer. The details were precise for mg gallic acid equivalent (GAE) per 100 g of a crude sample [21].

#### 2.3.3. Total Free Amino Acid Assessment 

The free amino acid substance of samples were determined by using Moore and Stein method (Spectrophotometer: ninhydrin solution and n-propanol at 570 nm absorbance) referred by [22].

#### 2.3.4. Grain Hardness Assay 

The Hardness of cooked rice used was measured by applying a texture analyzer (TA-XT2, Stable microsystems) with a 5 kg load cell [23]. The cooked sample of one kernel was directly situated on the inner cylindrical compressed probe with 100 mm diameter with a test speed of 0.5 mm/min.

#### 2.3.5. Total Protein Content Determination 

Protein was estimated by using the micro-Kjeldahl method and showed by way of total nitrogen × 5.95 g/100 g [24].

#### 2.3.6. Enzyme Interaction through Scanning Electron Microscopy (SEM)

SEM was utilized to observe the action of interaction enzymes with treated and untreated brown rice (JEOL-JSM 6610 LV, Japan; Plate No.18) using 15 kv electron voltage. The freeze-dried (5%) specimens were situated with two-layered adhesive tape fastened over metallic but sheeted with gold. 

### 2.4. Statistical Analysis

In addition to observational optimization, the enzyme treatment analysis turned out to be performed using multiple linear regression (MLR) and artificial neural network (ANN). The treatment variables (enzyme concentration, treatment time, temperature, and polishing time) influence quality attributes viz. mineral content (Ca, P, Fe). Phenolic content, free amino acid content, protein, and hardness was evaluated through multiple experimental designs. The multiple polynomial regression equations were used for the experimental design and produced to fit the experimental data; the applicable model terms are shown at Equation (1)

Y = b0 + b1X_1_ + b2X_2_ + b3X_3_ + b11X_12_ + b22X_22_ + b33 X_32_ + b12X_1_X_2_ + b13X_1_X_3_ + b23X_2_X_3_ (1)

Considering Y states expected inconsistent, b0, b1, b2, and b3 represents linear interval, b11, b22, and b33 portray quadratic gap, b12, b13, and b23 exists interlinkage interval, X_1_, X_2,_ and X_3_ indicates explanatory variables for enzyme processed rice. This statistical evaluation and analysis of variance were stated applying Design Expert Version 11.0 (Stat-Ease, Inc., Minneapolis, MN, USA). The significance exists at 0.01%, 1%, and 5% with the linear, cross-product, and square terms.

#### 2.4.1. Multiple Linear Regression (MLR)

The significant variables determining the target variable were chosen over the central composite rotatable design resultant in addition to being applied to create a multivariate analysis (MLR) equation implementing MATLAB’s fitlm function. A design elucidation was evaluated using the correlation coefficient (R^2^) and the sum of square error (SSE).

#### 2.4.2. Artificial Neural Network

**Figure 2:**
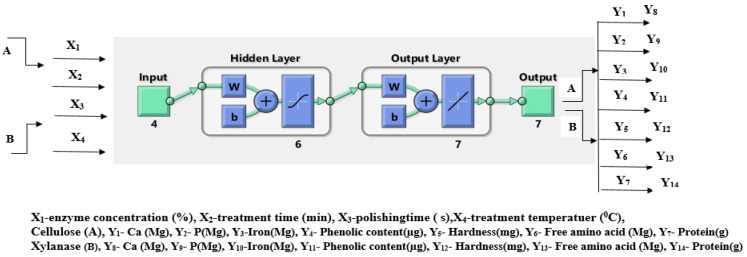
Configuration of multilayer ANN model with four input neurons, six hidden neurons and seven output neurons.

An ANN model consists of uncomplicated treatment components termed neurons that are interlinked with each other in a fuzzy logic configuration. A neuron receives a series of inputs that are filtered by an activation function to generate a primary output signal that serves as the stimulus for the next neuron. Training of the network is carried out by fine-tuning the progressive input neuron signals. MATLAB software R2018a was used for developing and testing the ANN design. The positive reaction neural network with a backpropagation algorithm comprising three strata, viz. an entry t layer, one concealed layer, and an exit layer, was employed as shown in Figure 2 below. The signals coming from the previous layer were processed, followed by transmission of output to the next layer on the basis of convergence criteria [13,25,26]. The variables selected for the input layer were cellulase—X_1_ X_2_ X_3_ X_4_ and xylanase—X_1_ X_2_ X_3_ X_4_, and the variables in the output layer were cellulase—Y_1_ Y_2_ Y_3_Y_4_ Y_5_ Y_6_ and xylanase—Y_7_ Y_8_ Y_9_ Y_10_ Y_11_ Y_12_ Y_13_ Y_14_ Y_15_. The input stratum included 4 neurons. The exit stratum comprised 6 neurons, whereas the number of neurons inside the concealed layer was optimized to be 7. The sigmoid transfer function “transit” was selected for activation of neurons at the hidden layer. For neurons of the exit layer, linear alienate operate “purelin” was utilized as this function is regarded as most suitable for backpropagation networks [25]. The Levenberg–Marquardt training algorithm was selected for training the network as this algorithm has now been calculated as the quickest technique to learn moderate-sized feed-forward neural networks until various hundred weights. Throughout fine-tuning, the actual observational data (30 runs) were been reproduced threefold (90 entries) and ruptured with three portions: 80:10:10 (%) for training, validation, and testing [25]. 

## 3. Results and Discussion

### 3.1. Multiple Linear Regression (MLR)

This research demonstrates the sustained findings by assessing and correlating details of appropriate models implementing central rotatable composite design. Multiple linear regression designs have been formulated and exploited to influence numerous predictor responses over measured variables. The design capability was measured through using correlation coefficient (r square), Fisher exact test (F), and Lack of fit (Table 1). This may indicate that significant variables affect every model for predicting its comparable variables (Table 2).

#### 3.1.1. Minerals

**Figure 3:**
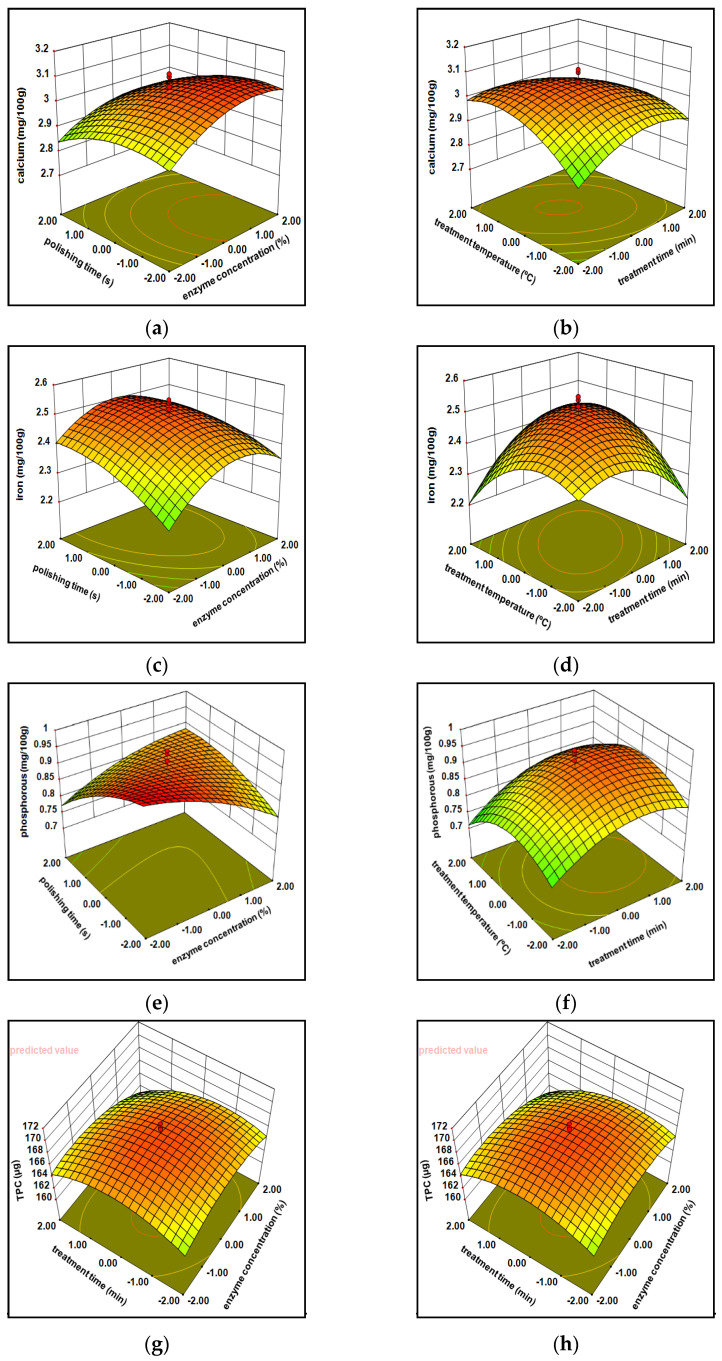

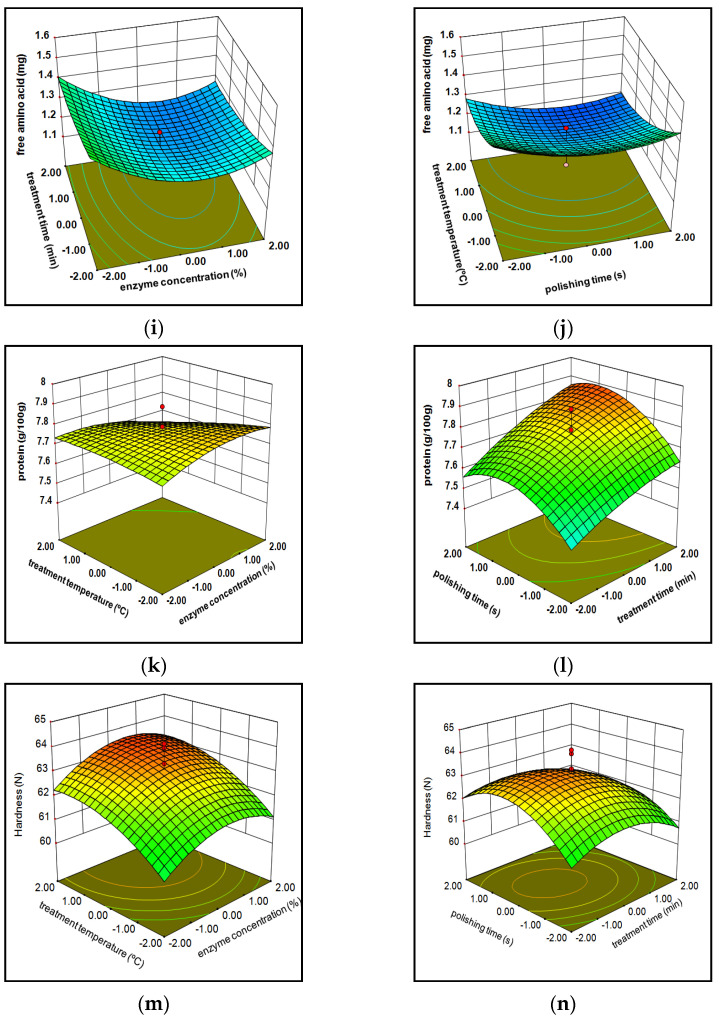
Effect of cellulase enzymatic treatment variables on calcium (**a**,**b**), iron (**c**,**d**), phosphorus (**e**,**f**), TPC (**g**,**h**), free amino acids (**i**,**j**), protein content (**k**,**l**), and hardness (**m**,**n**) of polished rice.

**Figure 4:**
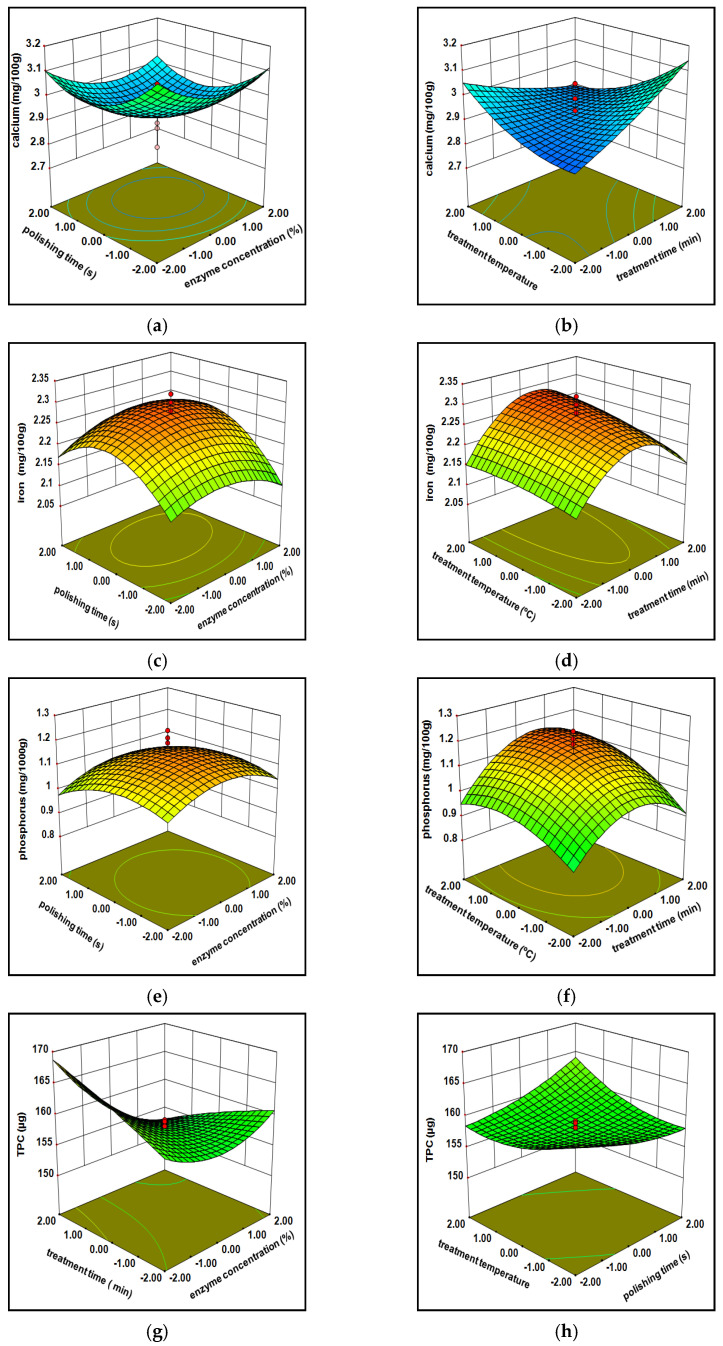

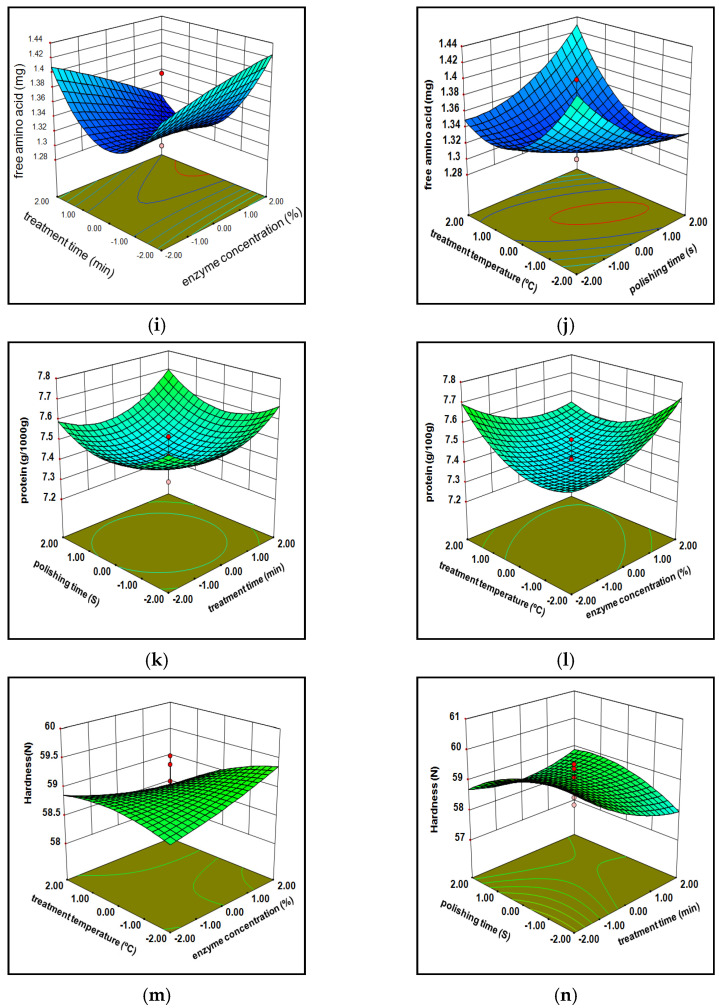
Effect of xylanase enzymatic treatment variables on calcium (**a**,**b**), iron (**c**,**d**), phosphorus (**e**,**f**), TPC (**g**,**h**), free amino acids (**i**,**j**), protein content (**k**,**l**) and hardness (**m**,**n**) of polished rice.

The cellulase-treated milled rice retained calcium fluctuated around 2.41 to 3.11 mg, whereas xylanase differed from 2.79 to 3.62 mg (Table 3). The maximum retention of calcium for cellulase rice was observed at 80% X_1_, 90 min X_2_, 30 s X_3_, 40 °C X_4_, and xylanase rice at 90% X_1_, 120 min X_2_, 20 s X_3_, and 35 °C X_4_. The lowest values of calcium retention of cellulase rice were found to be 70% X_1_, 120 min X_2_, 40 s X_3_, and 45 °C X_4_, and xylanase rice 80% X_1_, 90 min X_2_, 30 s X_3_, and 40 °C X_4_. The coefficient of determination (R^2^ = 89.38 (cellulase) and R^2^ = 90.73 (xylanase)) in terms of regression analysis was significant (<0.0001) as well as the lack of fit was nonsignificant. Figure 3a and Figure 4a) show the consequence of predictor parameters over mineral (Ca, P, Fe) retention about enzymatic milled rice. Figure 4a–f initially increased treatment temperature, and enzyme concentration up to 42.5 °C and 85% caused maximum retention of minerals (Ca, P, and Fe) in enzyme-treated bio-milled rice. After, losses of minerals occurred by continuously increasing temperature and enzyme concentration, whereas increased polishing time and treatment time continually caused better retention of minerals (concave shape) in cellulase rice and a reverse trend (convex shape) was observed for xylanase rice. The macronutrients existed inside the bran layer integrated to proteins; such distributions of minerals have been shown to be maximized in the exit stratum against endosperm [27]. Generally, during enzymatic treatment, the cellulase hydrolyzing enzyme acts on a coat around the cellulose [28]. It contains xylan and lignin; xylan invades the mean situation among the spathe of lignin remnants and intertwines through a covalent bond to the sheath in different positions. Covalent linkages around xylan through the lignin scabbard interweave with inter H-bonding imparts off a sheet nearby cellulosic manner. This cellulosic sheath is incorporated with macro elements and proteins. Initially, more enzyme concentration at low temperature causes breakages of covalent and inters H-bonding linkages and leads to moving macronutrients to the inner endosperm; this leads to more retained calcium in bio-polished rice at an initial level during polishing after losses have occurred due to enzyme inactivation by increasing temperature. This reason is due to phosphorus presence in the form of a phosphorus composite matrix with a cellulose stringy chain via hydrogen bonds and Van der Waals forces in the parallel direction. This cellulase reaction upon composite matrix leads to the creation of microfibrils; these were broad as well as forming a crystalline assemblage, leading to a significant release as water-soluble fragments from aleuronic stratum towards endosperm enriches the final products when enzyme concentrations are high its moves opposite to endosperm decreases the final product phosphorus concentration [19,20,29]. 

#### 3.1.2. Total Phenolic Content (TPC)

The observed value for the total phenolic content of cellulase milled rice varied between 151.92 to 169.66 µg, while xylanase milled varied within 139.78 to 176.89 µg. Correlation regression indicates that the phenolic content owned significantly (*p* < 0.001) was influenced via independent variables of cellulose as well as xylanase treated milled rice at a linear form and quadratic terms (Table 1 and Table 2). The mathematical formula generated with a variation in the phenolic content under individual parameters (X_1_, X_2_, X_3_, X_4_) was considerably suited within second-order polynomial equation (Equations (Y4) and (Y11) in Table 4). The high R^2^ = 0.87 and 0.87 (Table 1 and Table 2) data represent the acquaintance within viewing values and anticipated values and, as a result, improve the paradigm. (Figure 3g,h and Figure 4g,h) 3D surface plots show the effect of the whole phenolic substance against the independent variables. The phenolic importance of cellulase processed milled rice at the beginning increased arbitrarily (160 to 166.21 mg) after that moderately diminish (166.22 to 164.54 mg) demonstrates as a bulging (convex) shaped, continuous rising enzyme engrossment, time, and temperature, whereas xylanase treated milled rice shows a reverse trend in a concave way (initially decreases after that increases) with all the independent variables. Usually, phenolic acid concentrations are enhanced upon the endosperm towards aleuronic stratum. All phenols existed in an aleuronic layer derivate shape additionally connected to lignin and arabinoxylans compounds. These reactions validate that the enzymes induce the rupture of the attachment among lignin and cell walls and create lesser leaks around phenols derivatives: benzoic, gallic, protocatechuic, and vanillic acids in enzyme-processed milled rice throughout the treatment since phenolic compounds migrate towards the outer layer to endosperm.

#### 3.1.3. Free Amino Acids 

The free amino acid content of cellulase and xylanase milled rice ranging from 1.1 mg to 1.9 mg and 1.2 mg to 1.7 mg across the entire experimental considerations. The experimental and predicted values of xylanase are reported in Table 3. The best free amino acid was obtained by 70% X_1_,60 min X_2_, 20 s X_3_, 35 X_4_ and 80% X_1_,30 min X_2_, 30 s X_3_, 40 °C X_4_. An equilateral polynomial equation was adapted to both enzymes’ free amino acid practical information, which evolved a statistical retrogradation portrayed in Table 5 (Equations (Y6) and (Y13) in Table 4). The R^2^ (0.89 and 0.87), adjusted R^2^ (0.79 and 0.78), and the lack of fit was nonsignificant stated such that the fitness of model acts intimately among observational and anticipated values (Table 1 and Table 2). The free amino acid compound of cellulase (1.46 to 1.35 mg) and xylanase (1.41 to 1.33 mg) milled rice at first slightly diminished after they increased (1.21 to 1.42 mg) by an enhancement of all parameters (X_1_, X_2_, X_3_, X_4_) continuously from −2 to +2, observed in Figure 3i,j and Figure 4i,j. Free amino acid content is delicate for peak temperature over a lengthy period of cooking; it may cause loss of nutrients easily as a result of processing of the decreased enzyme losses through cooking; the ability of two enzymes in rupturing the glycosidic bonds among proteins with amino acids drives all the compounds (protein plus free amino acids) from the external stratum towards the internal stringy network endosperm. The glycosidic linkage associated compounds (proteins and amino acids) located at the bran layer were smoothly damaged in the middle of mechanical milling owing to the erratic mode through aleurone structure [2,30]. The previous statement reveals that cellulase processed brown rice, at first being slightly diminished, might be because cellulase performing over glycosidic bonds may have taken time. In contrast, xylanase immediately pretends as a result of being verified in the connected linkages (noncovalent bonds).

#### 3.1.4. Protein Composition

The protein compound values of cellulase and xylanase treated milled rice among 7.24 to 7.94 g and 7.29 to 8.16 g. The maximum protein content was shown at 80% X_1_, 150 min X_2_, 30 s X_3_, 40 °C X_4_ and 90%X_1_, 120 min X_2_, 30 s X_3_, 45 °C X_4_. A simplified equation (Equations (Y7) and (Y14) in Table 4) has been created regarding multinomial polynomial. It shows the result of significant parameters of protein recovery from milled rice. The correlation coefficient (R^2^) protein composition is nearly 95%, the F value was immensely significant (*p* < 0.0001), and the lack of fit was nonsignificant (*p* < 0.05) and may imply that the model has been confirmed within observational values and predicted values. The protein complex of cellulase processed rice was enhanced through the convex shape (progressively raised from 7.4 to 7.62 g, then moderately diminished until 7.56 g) by constantly increasing whole parameters (−2 to 2). In contrast, xylanase processed milled rice demonstrates slightly enhanced protein content over an increase of all parameters (Figure 3k,l and Figure 4k,l); this is the reason behind the large number of embryonic proteins tightly attached to a stringy network bran stratum via glycosidic bonds. While traditional milling processes a significant loss of protein due to the above reason, enzyme processing improves bond breakage, stringing of bonds and collisions among enzymes and its bonds lead to relocation of free amino acid outer matrix layer to inner endosperm during specific conditions [30]. 

#### 3.1.5. Hardness

The hardness of enzymes treated milled rice varied between 57.72 to 64.14 N and 56.45 to 61.78 N. The highest hardness of enzymatic milled rice was sustained at 80% X_1_, 90 min X_2_, 30s X_3_, 40 °C X_4_ (cellulase), and 90% X_1_, 60 min X_2_, 20s X_3_, 35 °C X_4_ (xylanase). The correlation coefficient (R^2^ = 89.9 and 88.6) for the regression model of the hardness of both enzymes treated milled rice was highly significant (<0.0001), and lack of fit has now been recognized as nonsignificant as evidenced in Table 1 and Table 2. Figure 3m,n and Figure 4m,n represents the hardness of cellulase processed milled rice, in the beginning, enhanced randomly after the moderate decline (60.51–60 N) by way of bulging shape (convex form) through enhancing the entire treatment of variables (−2 to 2), considering that xylanase milled rice shows linearly decreased hardness with all treatment conditions. This may be due to the aleuronic stratum removal caused by vulnerability around starch granules by enhanced gelatinization of the starch. Two of the maximum oil and fiber concentrations within the bran stratum inside the brown rice rendered a rigid structure with the hardness, subsequently reducing hardness continually by enzymes reacting over the Huron structure, thereby decreasing both compounds (fibers and oils) around the bran layer and making it smoother [21,31]. 

#### 3.1.6. Scanning Electron Microscopy Structural Analysis 

**Figure 5:**
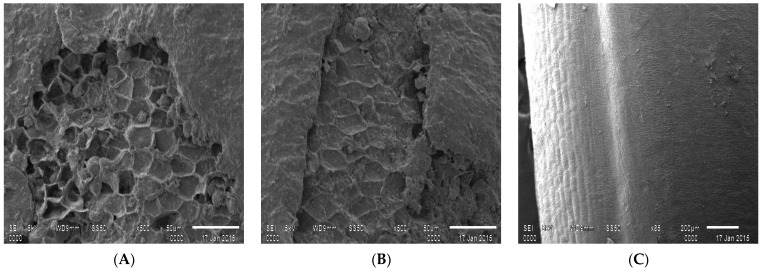
SEM images of cellulase treated rice (**A**), xylanase treated rice (**B**) and normal brown rice (**C**).

Microstructure analysis performed by SEM revealed loosening of rice bran from the endosperm due to the splitting activity of cellulase and xylanase on the bran layer of brown rice is observed in Figure 5A,B, which is deviated from regular brown rice followed in Figure 5C.

#### 3.1.7. Model Optimization 

Equation (1) shows the outcome of using multiple linear regression in the design of quadratic multinomial retaliation. Usually, many responses were used to produce the quadratic multinomial regression retaliation for predicting related responses. The models developed through significant variables have been reconstructed with MATLAB’s fitlm operation, as displayed in Table 4. It might have identified the novel linear equations that have shortened the significantly influenced parameters, and the achievement has been strengthened. Therefore, the present multivariate models (Table 4) have been endorsed as their potential for creating nutritionally enhanced enzymatic milled rice process parameters.

### 3.2. Artificial Neural Network 

**Figure 6:**
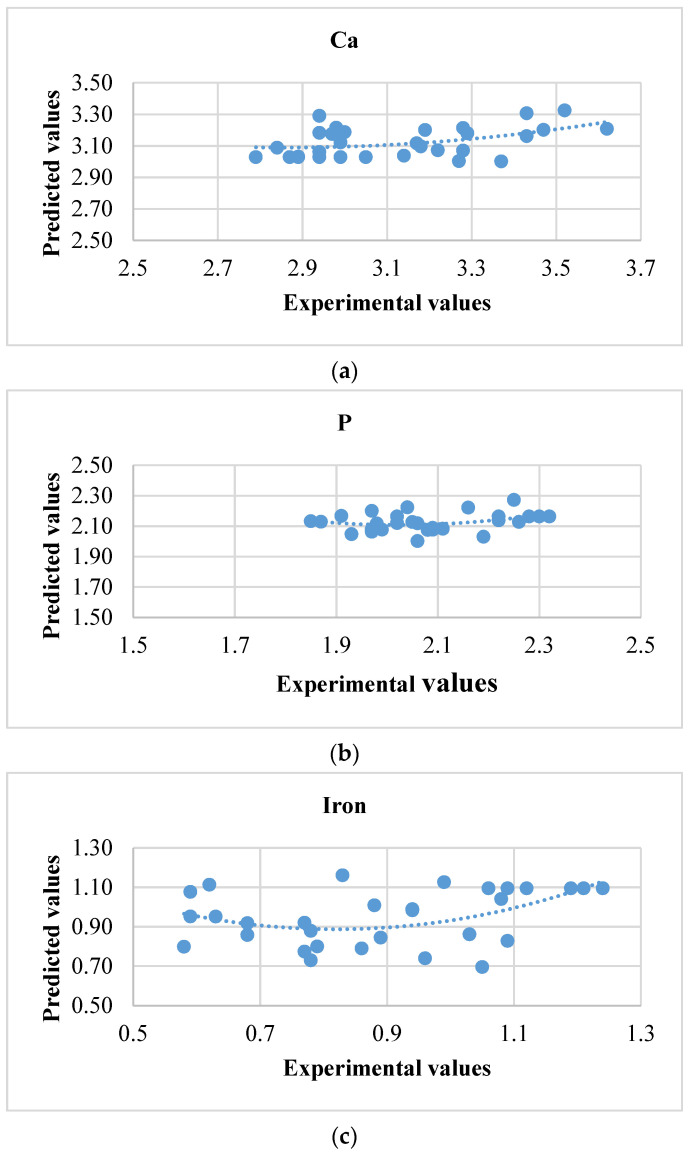

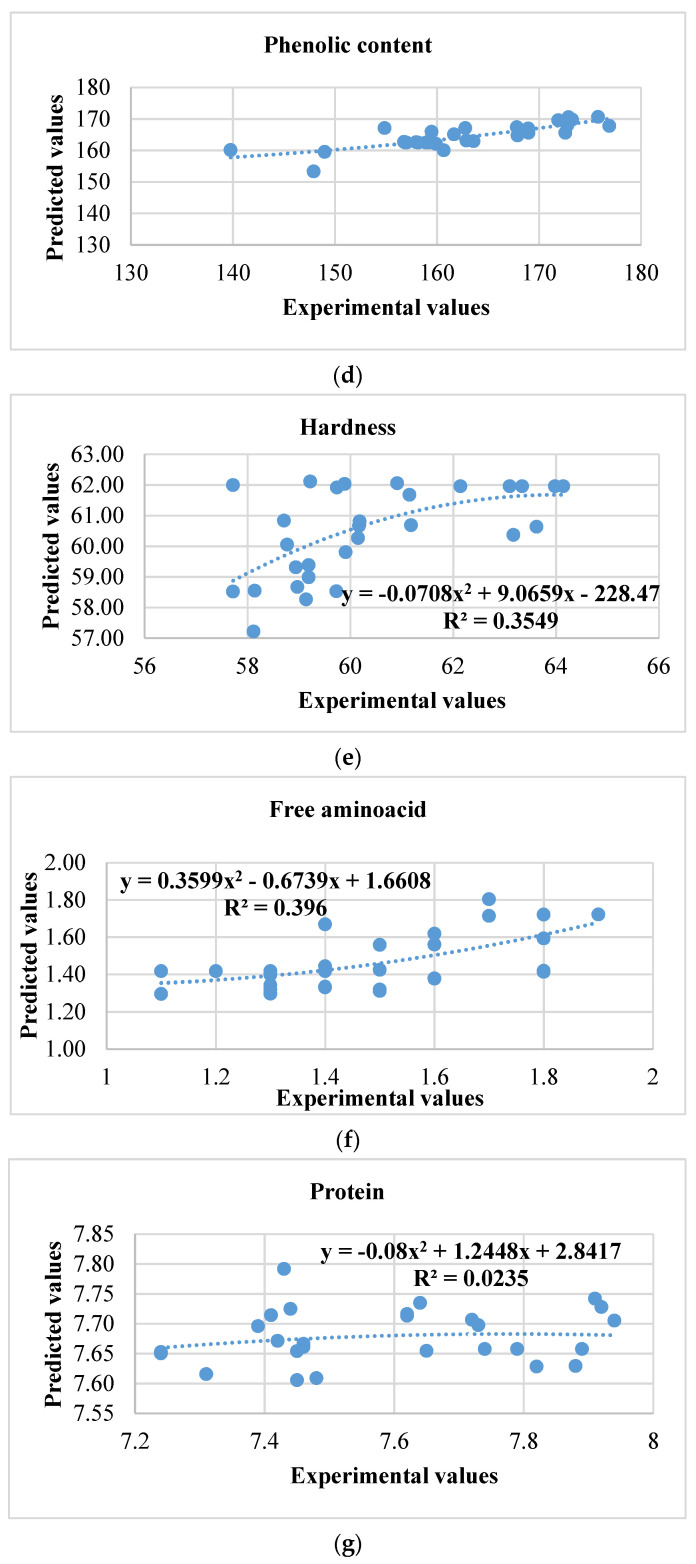
The relationship between experimental values and predicted values for the models developed using artificial neural network. (**a**) Calcium, (**b**) phosphorus, (**c**) iron, (**d**) phenolic content, (**e**) hardness, (**f**) free amino acid, and (**g**) protein of cellulase treated rice.

**Figure 7:**
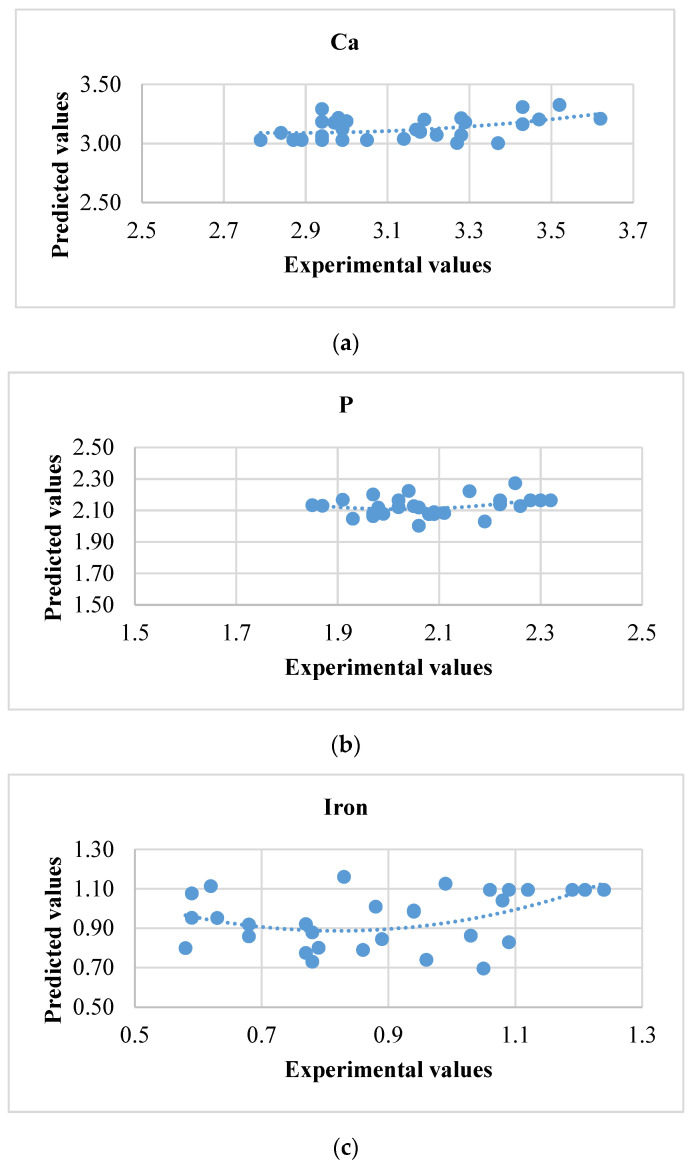

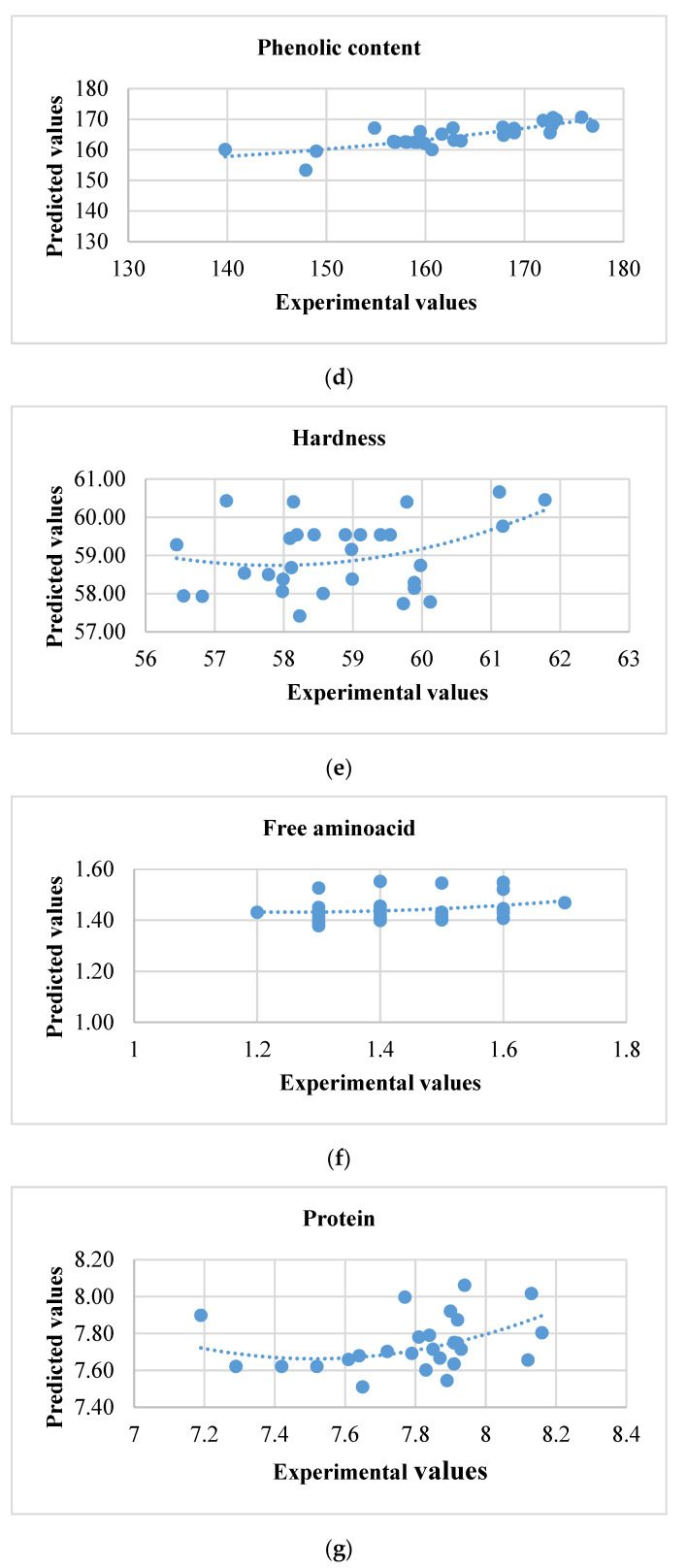
The relationship between experimental values and predicted values for the models developed using artificial neural network. (**a**) Calcium, (**b**) phosphorus, (**c**) iron, (**d**) phenolic content, (**e**) hardness, (**f**) free amino acid, and (**g**) protein of xylanase treated rice.

**Figure 8:**
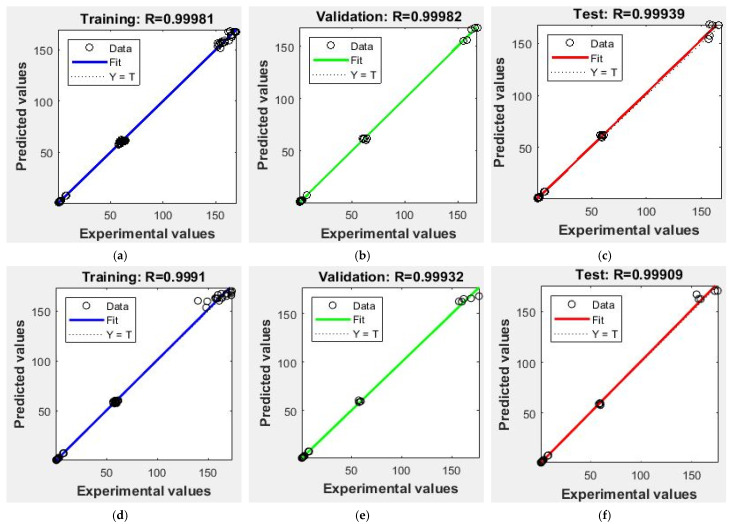
Regression plots for the training (**a**,**d**), validation (**b**,**e**) and (**c**,**f**) test for cellulose enzyme and xylanase treated milled rice ANN mode.

The ANN model was used further for differentiating the execution over the multiple linear regression model. The observed and anticipated assessment ranges were almost to an identical field (0.95 = 1) (Table 5). This interrelation distinguishes data and values expected as projected in Figure 6 and Figure 7. Counterpointing the previous exploration [26] comprises seven neurons at the exit structure while six neurons extracted at hidden stratum were abundant for establishing good achievement for ongoing investigations. Can it be ascertained that R^2^ enhanced over a maximum quantity of neurons at the invisible stratum. The complete artificial neural network design performance was better across multiple linear regression designs presented in Table 6. This might be noted that great fit would have been noticed wherein nutrients were retained in enzyme treated milled rice because determined R^2^ and MSE values fluctuated upon 0.91 to 0.97 and 0.005 to 6.13. The maximum R^2^ and minimum mean square error (MSE) values for ANN showed improved precision and authenticity [13,26]. ANN and MLR designs were advisable to anticipate the xylanase performance, which was superior over cellulase and stated R^2^ values of 0.97 and 0.90. Still, the ANN design could be strengthened through implementing maximum concealed neurons over every model; however, on this point, design engorgement is a possiblity. Therefore, a conclusion over quantity about neurons through established models has been applied appropriately. The MSE and correlation coefficient (R^2^) of a practiced network subsist approximately at 0.006 and 9.99. Attainment programs based on the aimed network, further to the scatter plot overtraining, validation, and test are depicted at Figure 8. Equating ANN and MLR, the ANN design achievement has been excellent; consequently, it is possible to exploit the speculation of the modeling of enzyme application for enhancing nutrients in cereals. 

#### Process Optimization

Economic software (Design-Expert version 11.0) was used for the optimization procedure for mathematical optimization for nutrients in the cellulase and xylanase processed milled rice. Simultaneously, enzyme concentration, treatment time, temperature, and milling time were the independent variables. The optimized values of processing conditions were acquired to create the following characteristics: maximum Ca, P, Fe, phenolic content, hardness, and free amino acid in enzyme processed polished rice. The desirable optimized values for cellulose (87.2% X_1_, 80.1 min X_2_, 21.8 s X_3_ and 33.95 °C) and xylanase (85.7% X_1_, 77.1 min X_2_, 20 s X_3_ and 35 °C X_4_) were found for treatment conditions in milled rice. The nutritional enhancement of enzyme treated milled rice under the above optimum conditions of experimental and predicted values can be observed in Table 6. Consequently, the chosen mathematical design exists precisely as well as immediately to create deviations in entire parameters.

## 4. Conclusions

The current research focused on modeling and optimizing process parameters for enhancing nutrients in enzyme treated milled rice by multiple optimization techniques. During the optimization of enzyme treatment, the consequences from the process assessment revealed that the enzyme concentration, treatment time, temperature, and polishing time had more impact upon the nutritional improvement of milled rice. The optimized cellulase treated milled rice was improved by 66% in calcium, 17% in iron, 64% in phosphorus, 78% in total phenol content, 33% in free amino acid, and 84% in protein content. In contrast, xylanase treated milled rice was improved by 70% in calcium, 15% in iron, 62% in phosphorus., 79% in total phenol content, 34% in free amino acid, and 83% in protein content compared to polished rice. The overall hardness (19–20%) of cooked milled rice was reduced. The xylanase showed better performance than cellulase. The designs were measured based upon the correlation coefficient (R^2^), the sum of squared error (SSE), and mean squared error (MSE). The observation outcome of MLR was enhanced by the multiple polynomial retrogression equations despite the predominate multilayer neuromorphic model of ANN obtained six neurons over a “transig” for activating in the hidden stratum. Two designs (MLR and ANN) have been better adapted to optimizing enzyme-treated milled functioning responses. The more significant coefficient and a lesser sum of squared error data of ANN indicate greater anticipation on observational values across MLR.

## Figures and Tables

**Table 1:** F-value, *p*-value and significance of each variable cellulase.

Source	Y_1_		Y_2_		Y_3_		Y_4_		Y_5_		Y_6_		Y_7_	
	F-Value	*p*-Value	F-Value	*p*-Value	F-Value	*p*-Value	F-Value	*p*-Value	F-Value	*p*-Value	F-Value	*p*-Value	F-Value	*p*-Value
Model	9.01	<0.0001	8.50	<0.0001	8.63	<0.0001	9.20	<0.0001	3.64260	<0.0001	8.90	<0.0001	7.84	0.0001
X_1_	0.07041	0.0075	0.01260	0.2267	0.00770	0.2088	3.64260	0.1694	4.10853	0.0505	0.09375	0.0089	0.00135	0.7220
X_2_	0.00426	0.4588	0.00453	0.4611	0.05133	0.0040	4.10853	0.1613	1.52510	0.0393	0.05041	0.0436	0.35526	<0.0001
X_3_	0.06406	0.0100	0.06100	0.0142	0.02870	0.0229	1.52510	0.0002	21.6030	0.1891	0.03375	0.0916	0.16666	0.0011
X_4_	0.01401	0.1884	0.00050	0.8043	0.00633	0.2521	21.6030	0.5067	17.1226	0.0001	0.35041	<0.0001	0.02801	0.1195
X_1_X_2_	0.13043	0.0740	0.24591	0.3154	0.01845	0.0053	17.1226	0.1079	19.5895	0.1063	0.38003	0.8096	0.05200	0.2365
X_1_X_3_	0.15003	0.4610	0.22682	0.3689	0.10607	0.0005	19.5895	0.0580	38.8484	0.2748	0.02503	0.1066	0.02234	0.0736
X_1_X_4_	0.07985	0.8637	0.04052	0.3978	0.02027	0.6338	38.8484	0.1733	9.23028	0.7490	0.12574	0.0433	0.38543	0.0880
X_2_X_3_	0.19914	<0.0001	0.28641	0.3978	0.14792	0.0281	9.23028	0.9077	2.37930	0.2093	0.08360	0.0061	0.00600	0.2950
X_2_X_4_	0.02722	0.0172	0.00855	0.0014	0.04730	0.5830	2.37930	0.7404	1.03530	0.5439	0.00062	0.0433	0.01562	0.0961
X_3_X_4_	0.00422	0.0822	0.00680	0.0013	0.08555	0.0029	1.03530	0.1137	0.08555	0.7326	0.03062	0.8096	0.03802	0.0805
X_1_^2^	0.00022	0.0008	0.00600	<0.0001	0.00105	0.0602	0.08555	0.0006	1.38650	0.0003	0.05062	<0.0001	0.03422	0.0399
X_2_^2^	0.2704	0.0004	0.00600	<0.0001	0.02640	0.0002	1.38650	<0.0001	0.31080	0.0002	0.10562	0.0977	0.0121	0.1610
X_3_^2^	0.0529	0.0050	0.12075	0.0391	0.00140	0.0501	0.31080	0.0336	0.09765	<0.0001	0.05062	0.0022	0.0324	<0.0001
X_4_^2^	0.0256	0.0001	0.12425	<0.0001	0.05640	<0.0001	0.09765	<0.0001	3.64260	<0.0001	0.00062	0.0081	0.0361	0.4566
R^2^	0.8938		0.8880		0.8896		0.8957		0.8902		0.8925		0.8798	
Adj. R^2^	0.7946		0.7835		0.7865		0.7883		0.7877		0.7946		0.7676	
Pred. R^2^	0.4490		0.4158		0.4418		0.4392		0.5148		0.4490		0.3695	
SSE	10.31		8.94		9.52		92.35		12.09		0.16		0.0183	
LOF	NS		NS		NS		NS		NS		NS		NS	

**Table 2:** F-value, *p*-value and significance of each variable.

Source	Y_8_		Y_9_		Y_10_		Y_11_		Y_12_		Y_13_		Y_14_	
	F-Value	*p*-Value	F-Value	*p*-Value	F-Value	*p*-Value	F-Value	*p*-Value	F-Value	*p*-Value	F-Value	*p*-Value	F-Value	*p*-Value
Model	10.49	<0.0001	10.52	<0.0001	8.78	<0.0001	7.21	1.316667	8.39	<0.0001	7.21	0.0002	6.17	0.0005
X_1_	0.03450	0.0718	0.00015	0.8351	0.00041	0.8261	349.988	−0.0125	0.01550	0.8452	0.00375	0.3243	0.01353	0.3871
X_2_	0.01870	0.1743	0.01306	0.0667	0.05226	0.0243	0.81770	−0.02083	4.51533	0.0040	0.01041	0.1101	0.03010	0.2039
X_3_	0.04593	0.0411	0.03081	0.0083	0.02041	0.1385	2.74050	−0.00417	0.09003	0.6390	0.00041	0.7388	3.75E-0	0.9632
X_4_	0.00700	0.3966	0.01126	0.0863	0.10401	0.0030	6.25260	0.004167	1.19260	0.1018	0.00041	0.7388	0.00220	0.7243
X_1_X_2_	0.18905	0.0426	0.02714	0.0765	0.15087	0.0212	283.930	−0.03125	0.07832	0.3036	0.01574	0.0551	0.17508	0.0056
X_1_X_3_	0.00771	0.8980	0.20503	0.2447	0.42287	0.8296	4.09425	0.04375	3.16491	0.1202	0.07145	0.0107	0.37000	0.0082
X_1_X_4_	0.31268	0.0028	0.12574	0.9322	0.10430	0.3400	32.8812	−0.01875	4.36802	0.1017	2.98E-0	0.2311	0.37000	0.0082
X_2_X_3_	0.07710	<0.0001	1.19E-0	0.0085	0.12190	0.0909	93.2305	−0.06875	0.54966	0.0046	0.01860	0.0004	0.36208	0.6128
X_2_X_4_	0.04515	0.0005	0.0121	0.4007	0.05522	0.2272	267.567	−0.00625	0.44555	0.0244	0.01562	0.6833	0.17850	0.2191
X_3_X_4_	0.00015	0.5921	0.0049	0.6114	0.0004	0.0007	13.9689	0.04375	1.06605	<0.0001	0.03062	0.0107	0.15800	0.0831
X_1_^2^	0.11730	0.0004	2.5E-05	0.0019	0.0081	0.0007	312.317	−0.00312	1.19355	0.6615	0.00562	0.7891	0.15800	0.0059
X_2_^2^	0.43230	0.3742	0.03062	<0.0001	0.02722	<0.0001	8.65830	0.071875	4.35765	0.0124	0.07562	<0.0001	0.00455	0.0003
X_3_^2^	0.17850	<0.0001	0.0025	<0.0001	0.01322	0.0030	579.726	0.021875	2.45705	0.0045	0.00062	0.0759	0.02805	0.0003
X_4_^2^	0.00275	0.0111	0.0009	0.3697	0.1521	0.0017	99.9500	0.046875	21.3213	0.2552	0.03062	0.0010	0.05880	0.0003
R^2^	0.9073		0.9076		0.8913		0.8706		0.8867		0.8706		0.8574	
Adj. R^2^	0.8209		0.8214		0.7898		0.7499		0.7810		0.7499		0.7243	
Pred. R^2^	0.5896		0.5421		0.4708		0.3438		0.4650		0.3438		0.2429	
SSE	0.14		0.050		0.13		203.85		5.89		8.69		6.44	
LOF	NS		NS		NS		NS		NS		NS		NS	

**Table 3:** ANN design (experimental and predicted values) for performance of xylanase.

Y_8_		Y_9_		Y_10_		Y_11_		Y_12_		Y_13_		Y_14_	
Exp.	Pred.	Exp.	Pred.	Exp.	Pred.	Exp.	Pred.	Exp.	Pred.	Exp.	Pred.	Exp.	Pred.
2.84	3.09	2.06	2.00	0.94	0.98	160.67	160.03	61.12	60.66	1.50	1.41	7.65	7.51
2.94	3.18	1.93	2.05	0.77	0.92	162.78	167.10	61.78	60.45	1.40	1.45	7.91	7.75
3.47	3.20	1.97	2.06	0.86	0.79	172.89	170.53	58.11	58.68	1.60	1.41	7.61	7.66
3.62	3.21	2.05	2.13	1.09	0.83	168.95	166.97	59.98	58.73	1.50	1.43	7.92	7.87
3.22	3.07	2.08	2.08	0.59	0.95	147.92	153.31	58.09	59.44	1.30	1.38	7.91	7.63
3.14	3.04	1.98	2.12	0.62	1.11	157.97	162.59	58.14	60.40	1.60	1.44	7.83	7.60
3.00	3.19	1.99	2.08	0.58	0.80	176.89	167.76	57.43	58.53	1.40	1.40	7.64	7.68
3.18	3.10	2.16	2.22	0.63	0.95	162.89	163.10	56.55	57.94	1.30	1.41	8.16	7.80
3.43	3.31	2.09	2.08	0.78	0.73	173.24	169.78	58.57	58.00	1.40	1.55	7.77	8.00
2.98	3.21	2.06	2.12	0.68	0.86	159.47	165.93	57.99	58.37	1.30	1.53	7.9	7.92
3.43	3.16	2.02	2.16	1.03	0.86	172.78	167.95	56.82	57.93	1.60	1.43	7.81	7.78
3.27	3.00	1.97	2.20	0.99	1.13	139.78	160.10	56.45	59.27	1.40	1.43	8.12	7.66
3.52	3.32	1.97	2.08	1.05	0.69	175.78	170.64	59.73	57.73	1.60	1.55	8.13	8.02
3.19	3.20	2.02	2.12	0.78	0.88	168.98	165.59	57.78	58.50	1.60	1.52	7.19	7.90
2.97	3.18	2.22	2.14	0.89	0.84	171.89	169.60	59.89	58.14	1.40	1.43	7.92	7.75
2.89	3.03	2.25	2.27	1.08	1.04	148.99	159.52	60.12	57.78	1.40	1.41	7.84	7.79
3.29	3.18	2.09	2.09	0.79	0.80	172.59	165.58	58.99	58.37	1.30	1.39	7.72	7.70
3.17	3.12	2.11	2.08	0.88	1.01	167.89	164.75	59.78	60.39	1.30	1.45	7.79	7.69
2.94	3.06	1.85	2.13	0.59	1.08	156.78	162.69	61.17	59.76	1.70	1.47	7.87	7.67
2.99	3.12	1.91	2.17	0.68	0.92	154.87	167.09	59.89	58.29	1.50	1.41	7.93	7.71
3.37	3.00	1.87	2.13	0.83	1.16	159.91	162.07	57.17	60.43	1.40	1.44	7.89	7.54
3.28	3.07	2.04	2.22	0.94	0.99	163.59	162.91	57.98	58.05	1.40	1.41	7.91	7.75
3.28	3.21	2.19	2.03	0.77	0.77	161.67	165.10	58.98	59.15	1.50	1.40	7.85	7.71
2.94	3.29	2.26	2.13	0.96	0.74	167.82	167.39	58.23	57.41	1.50	1.55	7.94	8.06
3.05	3.03	2.3	2.16	1.12	1.09	159.23	162.47	58.44	59.54	1.40	1.43	7.52	7.62
2.99	3.03	2.32	2.16	1.06	1.09	156.9	162.47	59.11	59.54	1.30	1.43	7.42	7.62
2.87	3.03	2.22	2.16	1.21	1.09	158.92	162.47	59.54	59.54	1.60	1.43	7.42	7.62
2.94	3.03	2.28	2.16	1.19	1.09	157.01	162.47	59.40	59.54	1.40	1.43	7.42	7.62
2.89	3.03	2.3	2.16	1.24	1.09	158.15	162.47	58.19	59.54	1.50	1.43	7.42	7.62
2.79	3.03	2.22	2.16	1.09	1.09	156.92	162.47	58.89	59.54	1.20	1.43	7.29	7.62

**Table 4:** Quadratic models developed using independent variables from enzymatic treatment experimental design.

Response	Equation
CellulaseY_1_	Y_1_ = 3.05 + 0.0547X_1_ + 0.0246X_4_ + 0.041X_1_X_2_
Y_2_	Y_2_ = 0.92 + 0.054X_1_X_2_ + 0.073X_1_X_3_ + 0.041X_2_X_3_ + 9.375 × 10^−3^ X_2_X_4_
Y_3_	Y_3_ = 2.49 + 0.0237X_1_ + 0.014X_2_ + 0.050X_3_ + 4.583 × 10^36^ X_4_ + 0.019X_1_X_4_ + 0.019X_2_X_3_ + 0.087X_2_X_4_
Y_4_	Y_4_ = 168.24 + 1.27X_1_X_3_ + 0.89X_1_X_4_ + 1.04X_3_X_4_
Y_5_	Y_5_ = 63.14 + 0.39X_1_ + 0.25X_3_ + 0.95X_4_ + 0.25X_1_X_3_ + 0.073X_1_X_4_
Y_6_	Y_6_ = 1.23 − 0.063X_1_ + 0.044X_1_X_3_ + 0.056X_1_X_4_ + 0.081X_2_X_3_ + 0.056X_2_X_4_ + 0.12X_1_^2^ + 0.034X_2_^2^ + 0.072X_3_^2^ + 0.059X_4_^2^
Y_7_	Y_7_ = +7.77 + 0.12X_2_ + 0.083X_3_ + 0.031X_1_X_2_ + 0.027 X_2_X_3_ + 0.047 X_3_X_4_
Xylanase Y_8_	Y_8_ = 2.92 + 0.028X_2_ + 0.053X_1_X_2_ + 3.125 × 10^−0.086^ X_1_X_3_ + 0.083X_1_^2^ + 0.017X_2_^2^ + 0.11X_3_^2^ + 0.053X_4_^2^
Y_9_	Y_9_ = 2.27 + 2.500 × 10^−3^ X_1_ + 0.023X_2_ + 0.036X_3_ + 0.022X_4_ + 0.028X_1_X_2_ + 0.018X_1_X_3_ + 0.044X_2_X_3_ + 0.013X_2_X_4_ + 7.500 × 10^−3^ X_3_X_4_
Y_10_	Y_10_ = 1.15 + 4.167 × 10^−3^ X_1_ + 0.047X_2_ + 0.066X_4_ + 0.059X_1_X_2_ + 5.000 × 10^−3^ X_1_X_3_ + 0.029X_2_X_4_ + 0.098X_3_X_4_
Y_11_	Y_11_ = 168.24 + 1.27X_1_X_3_ + 0.89X_1_X_4_ + 1.04X_3_X_4_
Y_12_	Y_12_ = 63.14 + 0.39X_1_ + 0.25X_3_ + 0.95X_4_ + 0.073X_1_X_4_
Y_13_	Y_13_ = 1.23 + 0.044X_1_X_3_ + 0.056X_1_X_4_ + 0.081X_2_X_3_ + 0.12X_1_^2^ + 0.034X_2_^2^ + 0.072X_3_^2^ + 0.059X_4_^2^
Y_14_	Y_14_ = 7.77 − 7.500 × 10^−3^ X_1_ + 0.12X_2_ + 0.083X_3_ − 0.034X_4_ − 0.044 X_1_^2^ + 0.031 X_1_X_2_ + 0.027 X_2_X_3_ + 0.047 X_3_ X_4_

**Table 5:** ANN design (experimental and predicted values) for performance of cellulase.

Y_1_		Y_2_		Y_3_		Y_4_		Y_5_		Y_6_		Y_7_	
Exp.	Pred.	Exp.	Pred.	Exp.	Pred.	Exp.	Pred.	Exp.	Pred.	Exp.	Pred.	Exp.	Pred.
2.51	2.73	2.04	2.05	0.95	0.79	165.33	163.84	58.14	58.55	1.90	1.72	7.45	7.61
2.65	2.72	2.12	2.06	0.58	0.75	162.88	159.21	59.73	58.53	1.70	1.71	7.31	7.62
2.82	2.73	1.93	2.05	0.76	0.78	165.67	162.60	57.72	58.52	1.80	1.72	7.48	7.61
2.96	2.75	1.82	2.11	0.71	0.74	158.9	157.41	59.19	58.99	1.40	1.67	7.82	7.63
2.88	2.82	2.29	2.19	0.64	0.74	154.91	156.28	58.94	59.32	1.60	1.62	7.46	7.67
2.66	2.87	2.33	2.27	0.67	0.72	156.74	153.89	59.91	59.81	1.50	1.56	7.39	7.70
2.45	2.88	2.25	2.26	0.71	0.75	154.23	157.42	59.19	59.39	1.80	1.59	7.73	7.70
2.76	2.86	2.15	2.25	0.97	0.68	152.21	153.14	58.97	58.67	1.60	1.56	7.91	7.74
2.79	2.91	1.99	2.35	0.91	0.86	160.11	167.67	59.22	62.11	1.80	1.41	7.24	7.65
2.89	2.85	2.22	2.31	0.71	0.80	164.21	163.78	61.15	61.68	1.60	1.38	7.42	7.67
2.92	2.89	2.25	2.33	0.95	0.83	162.7	166.00	59.74	61.92	1.30	1.40	7.46	7.66
3.01	2.73	2.28	2.21	0.79	0.66	155.32	154.87	61.18	60.69	1.30	1.30	7.41	7.71
2.92	2.78	2.05	2.24	0.56	0.71	156.21	158.58	60.18	60.82	1.40	1.33	7.72	7.71
2.91	2.74	1.95	2.21	0.47	0.66	158.9	156.05	63.17	60.37	1.50	1.31	7.44	7.72
2.41	2.75	2.19	2.21	0.59	0.67	151.92	156.63	60.15	60.27	1.30	1.32	7.92	7.73
2.54	2.76	2.25	2.22	0.78	0.67	157.29	157.67	58.77	60.06	1.40	1.33	7.64	7.73
2.65	2.91	2.08	2.35	0.81	0.86	163.67	168.05	59.89	62.04	1.80	1.42	7.65	7.65
2.96	2.75	2.29	2.23	0.79	0.68	157.21	156.95	60.17	60.67	1.50	1.32	7.62	7.71
2.78	2.91	2.15	2.35	0.57	0.86	157.21	167.93	60.91	62.06	1.30	1.42	7.45	7.65
2.79	2.79	2.25	2.25	0.74	0.72	158.78	159.42	58.71	60.84	1.30	1.34	7.94	7.71
2.92	2.90	2.31	2.34	0.76	0.86	165.87	167.79	57.72	62.00	1.50	1.42	7.24	7.65
2.81	2.87	2.51	2.25	0.83	0.74	162.44	167.24	59.14	58.27	1.40	1.44	7.43	7.79
2.77	2.68	2.19	2.00	0.65	0.68	154.92	151.73	58.12	57.22	1.70	1.80	7.88	7.63
2.71	2.72	2.12	2.21	0.57	0.65	152.89	154.64	63.62	60.64	1.10	1.30	7.62	7.72
3.1	2.91	2.54	2.35	0.96	0.86	165.96	167.90	62.14	61.96	1.30	1.42	7.89	7.66
2.96	2.91	2.55	2.35	0.85	0.86	169.01	167.90	63.34	61.96	1.20	1.42	7.79	7.66
3.11	2.91	2.41	2.35	0.97	0.86	167.89	167.90	63.1	61.96	1.30	1.42	7.74	7.66
3.05	2.91	2.49	2.35	0.94	0.86	168.11	167.90	63.98	61.96	1.40	1.42	7.74	7.66
3.03	2.91	2.52	2.35	0.91	0.86	169.66	167.90	64.14	61.96	1.20	1.42	7.74	7.66
3.06	2.91	2.45	2.35	0.87	0.86	168.79	167.90	62.14	61.96	1.10	1.42	7.74	7.66

**Table 6:** Performance measure of artificial neural network model.

Variables	Experimental Range	Predicated Range	R^2^	MSE
Cellulase Y_1_	2.41–3.11	2.68–2.91	0.91	1.1641
Y_2_	1.82–2.51	2.00–2.35	0.94	1.1622
Y_3_	0.47–0.97	0.65–0.87	0.96	6.1344
Y_4_	152.21–169.66	151.92–168.05	0.92	0.971
Y_5_	57.72–64.14	57.72–64.14	0.94	2.734
Y_6_	1.10–1.90	1.30–1.80	0.93	4.924
Y_7_	7.24–7.88	7.62–7.72	0.96	0.229
Xylanase Y_8_	2.84–3.62	3.00–3.32	0.91	0.677
Y_9_	1.93–2.32	2.00–2.27	0.94	0.354
Y_10_	0.58–1.24	0.69–1.16	0.93	0.005
Y_11_	147.92–176.89	159.52–170.64	0.90	1.667
Y_12_	57.45–61.78	57.41–60.66	0.95	2.871
Y_13_	1.20–1.70	1.39–1.55	0.92	0.195
Y_14_	7.29–8.16	7.51–8.02	0.97	0.2044

## Data Availability

Not applicable.
